# *Candida glabrata* meningitis in a patient with newly diagnosed acquired immunodeficiency syndrome from Sikkim, India

**DOI:** 10.22034/CMM.2024.345242.1542

**Published:** 2024-05-07

**Authors:** Sunu Hangma Subba, Tara Devi Sharma, Yeshi Palden Dopthapa, Ugen Gyatso N Bhutia, Rekha Sharma

**Affiliations:** 1 Department of Clinical Microbiology, Sir Thutob Namgyal Memorial Hospital, Sochakgang, Gangtok, India; 2 Department of Medicine, Sir Thutob Namgyal Memorial Hospital, Sochakgang, Gangtok, India; 3 Department of Microbiology, Sikkim Manipal Institute of Medical Science, Gangtok, India

**Keywords:** Acquired immune deficiency syndrome, Candidemia, *Candida glabrata*, Central nervous system infections, Fungal meningitis

## Abstract

**Background and Purpose:**

*Candida* infections in India have shifted, with an increase in the incidence rate of invasive candidiasis, particularly due to non-*albicans* species. The central nervous system infections by *Candida glabrata* are sparsely reported and more understanding and research is needed regarding these infections.

**Case report:**

This study reported an unusual case of *C. glabrata* meningitis in a middle-aged female with pulmonary tuberculosis and newly diagnosed acquired immunodeficiency syndrome with a low cluster of differentiation 4 count (12 cells/mm^3^). Initially, the patient was treated with fluconazole. Subsequently, the patient underwent therapy involving amphotericin B and flucytosine. The cerebrospinal fluid cultures eventually grew *C. glabrata*, confirmed by matrix-assisted laser desorption ionization time-of-flight analysis. Despite switching to amphotericin B and flucytosine, the conditions of the patient deteriorated, leading to her death.

**Conclusion:**

*Candida glabrata* candidemia requires meticulous and vigilant management due to its high mortality rate and relatively higher resistance to azoles, particularly fluconazole. This case underscored the severe and pressing challenges in the management of *C. glabrata* meningitis, particularly in immunocompromised patients.

## Introduction

With the increase in candidemia cases, a change in the spectrum of *Candida* species has been noticed in major hospitals in India. Non-*albicans Candida* species are isolated from 30-90% of cases of invasive candidiasis. In contrast to *C. parapsilosis* or *C. glabrata*, *C. tropicalis* is the most common species among non-*albicans Candida* isolates [ 1 ]. Meningeal infection due to *C. dubliniensis*, *C. tropicalis*, and *C. glabrata* have all been described, yet *C. albicans* remains the leading cause of *Candida* infections [ 2 , 3 , 4 ]. *Candida glabrata* infections can affect mucosal surfaces or become systemic, particularly in individuals with compromised immune systems or conditions, such as diabetes mellitus. Unlike other *Candida* species, *C. glabrata* does not exhibit dimorphism and remains in the blastoconidia form, acting as a commensal organism or a pathogen. Treatment of *. glabrata* infections is challenging due to their frequent resistance to many azoles, especially fluconazole [ 5 ]. This case study detailed a rare occurrence of *C. glabrata* isolated from the cerebrospinal fluid (CSF) of a patient newly diagnosed with AIDS and pulmonary tuberculosis.

## Case report

An Asian woman in her middle age arrived at the facility with the complaint of severe headaches persisting for a week, along with decreased appetite and weakness. She had a history of pulmonary tuberculosis, for which she had completed treatment two months before her admission.

On day 0 of examination, the patient was conscious but exhibited irritability. Neurological evaluation revealed a Glasgow Coma Scale score of 15/15 and mild neck stiffness. Routine laboratory tests revealed a raised C-reactive protein level of 15.13 mg/L (normal range: <10 mg/L), an elevated neutrophil count of 91%, and an increased ESR of 105 mm in the first hour (normal range: <20 mm/h). Liver and kidney function tests were within normal limits. Hepatitis B and C were negative based on the immunochromatographic method. Additionally, the Widal test for enteric fever and the rapid plasma reagin test for syphilis also came out negative.

However, a plain computed tomography (CT) scan of the brain showed focal calcified lesions measuring 9 × 8 mm in the left parietal lobe, suggesting calcified granuloma or other calcified lesions. Treatment started with parenteral broad-spectrum antibiotics, analgesics, and mannitol 20% due to the history of the patient in terms of severe week-long headaches and a CT scan results showing calcified lesions in the brain. It should be mentioned that increased intracranial pressure or other intracranial pathology was suspected. Moreover, the CT thorax report indicated likely sequelae of pulmonary tuberculosis with potential active disease. Considering the history of the patient regarding pulmonary tuberculosis and her present neurological symptoms, tuberculous meningitis was included as a differential diagnosis. Elevated erythrocyte sedimentation rate (ESR) and C-reactive protein levels further supported the presence of an inflammatory process (Table 1).

**Table 1:** List of relevant laboratory and radiology findings of fungal Meningitis caused by *Candida glabrata*

**Routine laboratory tests**	Neutrophil count (91%)	Raised
	Erythrocyte sedimentation rate	Raised
	C-reactive protein	Raised
	Blood sugar	Normal
	Total protein	Normal
**Serology**	HIV-1	Positive
	CD4 count	12 cells/mm^3^
**Radiology**	Brain CT scan (plain)	Focal calcified lesions, 9×8 mm, left parietal lobe, suggesting calcified granuloma/other calcified lesions
	Thorax CT (contrast)	Likely sequelae of pulmonary tuberculosis with potential active disease.
	Brain MRI	5×5 mm hypointense focus, left posterior parietal regions, no significant perifocal edema, suggesting healed granuloma
**CSF examination**	WBC	15 cells/mm^3^
	Protein	35 mg/dl
	Glucose	43 mg/dl
	LDH	23 units/L
	ADA (Adenosine deaminase)	11 units/L
	ZN Stain	No acid-fast bacilli observed
	India ink	Round yeast cells with no clear capsule around the cell
	CBNAAT	No MTB detected
**Sputum**	ZN stain	No Acid-fast bacilli observed
	CBNAAT	No MTB detected
**Pathology**	Few lymphocytes and numerous yeasts-like bodies on the smear.	
**CSF fungal culture**	Sabouraud dextrose agar	Whitish moist colonies after three days of incubation at 37 °C
	CHROM agar (Hi-Media)	Purplish color growth
	Urease and Germ tube tests	Negative
	Gram stains	Gram-positive yeast cells
**Identification**	MALDI-TOF analysis revealed that the organism was *Candida glabrata*	
**Antifungal susceptibility testing**	**Antifungal disks on Mueller-Hinton Agar + 2% Glucose and 0.5 µg/mL methylene blue dye medium**	
	Amphotericin B 20 mcg	Sensitive
	Itraconazole 10 mcg	Sensitive
	Fluconazole 25 mcg	Sensitive
	Voriconazole 1 mcg	Resistant
	**Vitek 2 Report (MIC)**	
	Amphotericin B	2 µg/ml (resistant)
	Flucytosine	2 µg/mL (resistant)
	Vitek 2 could not provide the MIC for other antifungals, such as fluconazole, itraconazole, and voriconazole	

MALDI-TOF: matrix-assisted laser desorption ionization time-of-flight, CD4: cluster of differentiation 4, CT: computed tomography, WBC: white blood cells, LDH: lactate dehydrogenase, ZN: Ziehl-Neelsen, CBNAAT: cartridge-based nucleic acid amplification test, MRI: magnetic resonance imaging, CSF: cerebrospinal fluid, ADA: adenosine deaminase, MTB: mycobacterium tuberculosis, MIC: minimum inhibitory concentration

On day 1 after the performance of a fundus examination, a lumbar puncture was conducted. The CSF analysis revealed 15 white blood cells per mm³ (predominantly lymphocytes), glucose at 43 mg/dl, a protein level of 35 mg/dl, lactate dehydrogenase at 23 units/L, and adenosine deaminase at 11 units. Blood sugar was 158 mg/dL and the total protein was 7.3 g/dL (normal range: 6.4-8.3 g/dL). The CSF findings were consistent with a mild inflammatory response with lymphocytic predominance and relatively normal protein and glucose levels. The Ziehl-Neelsen stain of the CSF showed no acid-fast bacilli. The India ink preparation from the uncentrifuged CSF sample displayed round yeast cells but lacked a clear capsule around them. The CSF for cartridge-based nucleic acid amplification test for mycobacterium tuberculosis (MTB) reported no MTB. Based on CSF findings, fungal meningitis was suspected, and parenteral fluconazole 400 mg every 24 h and steroid (dexamethasone 8 mg IV) every 12 h were administered. 

On day 3, the patient experienced two episodes of generalized seizures; hence, parenteral phenytoin 100 mg IV every 8 h and levetiracetam 500 mg 12 h orally were administered. On day 4, the condition of the patient remained the same, with no seizure episodes, but the headache was persistent, and she remained irritable. The patient tested positive for HIV-1, initially screened using the enzyme immunoassay method (Combaids) and subsequently confirmed by two other tests, namely immunochromatography (Standard Q) and an immune filtration method (UltraDot), following the National AIDS Control Organization testing guidelines. The cluster of differentiation 4 (CD4) count reported 12 cells/mm^3^ by Pima CD4 Analyzer. The CSF culture on Sabouraud dextrose agar with gentamicin produced whitish, moist colonies after three days of incubation at 37 °C. Furthermore, Urease and germ tube tests were negative, and CHROM agar (Hi-Media) showed purplish color growth.

On day 5, the report of antifungal susceptibility testing (AFST) using antifungal disks on Mueller-Hinton Agar + 2% Glucose and 0.5 µg/mL Methylene Blue Dye Medium (GMB, Himedia) was interpreted. The testing involved antifungal discs (Microexpress) of amphotericin B 20 mcg, itraconazole 10 mcg, fluconazole 25 mcg, and voriconazole 1 mcg. The results revealed that the fungus was sensitive to amphotericin B, fluconazole, and voriconazole. However, the yeast isolate was resistant to itraconazole. Based on the AFST report, intravenous deoxycholate amphotericin B 50 mg and oral flucytosine 500 mg every 6 h were administered, and fluconazole was discontinued. The patient could not afford liposomal amphotericin B treatment due to financial constraints. On the same day, brain magnetic resonance imaging reported a 5×5 mm hypointense focus on all left posterior parietal regions with no significant perifocal edema, suggesting healed granuloma.

From day 6 onwards, the condition of the patient started deteriorating. After 7 days of incubation, blood cultures for fungal infection tested negative. On day 8, the medical team observed complications, including mild kidney function abnormalities and hypokalemia. However, the condition of the patient deteriorated gradually, marked by a decline in consciousness. Finally, she passed away on day 9 of her hospitalization.

The laboratory lacked the capability to identify the fungal pathogen; therefore, a month later, matrix-assisted laser desorption ionization time-of-flight (MALDI-TOF) analysis revealed that the organism was *C. glabrata*. According to the Vitek 2 report, the MIC for amphotericin B and flucytosine were 2 and 2 µg/mL, respectively. However, Vitek 2 could not provide the MIC for other antifungals, such as fluconazole, itraconazole, and voriconazole. Based on microbiological findings, the organism was identified as *C. glabrata*.

## Discussion

*Candida glabrata*, once regarded as non-pathogenic, is now increasingly recognized as a cause of various infections. Each year, these infections become more frequent and challenging to treat, particularly in patients with any degree of immunosuppression. [ 6 ]. *Candida glabrata* infections are uncommon; hence, no randomized controlled trials are available to assess the correct selection and duration of antifungal therapy. Table 2 shows the few published case studies on meningitis caused by *C. glabrata* [ 7 , 8 , 9 ].

**Table 2:** Published documented cases of meningitis caused by *Candida glabrata*

Reference	Clinical details	Diagnosis	Treatment	Outcome
**Colomba et al. 2014 [ 7 ].**	An unusual case of *C. glabrata* meningitis and endocarditis in a young Caucasian female with a prosthetic aortic valve who suffered from a dissecting thoracoabdominal aortic aneurysm highlights the intrinsic resistance of *C. glabrata* to azoles.	*Candida glabrata* was isolated from the CSF culture. To assess the sensitivity to antifungals, a broth colorimetric microdilution method was used (Fungifast AFG) that allowed susceptibility testing to five antifungals.	Liposomal amphotericin B (3 mg/kg/daily) for 4 weeks and then oral fluconazole 400 mg/daily was administered.	Recovered
**Zhu et al. 2018 [ 8 ].**	A 25-year-old female with multiple brain abscesses. The patient was initially treated with antibiotics; however, 2 months after the initial treatment, her condition deteriorated and she became vegetative. Following transfer to the China-Japan Union Hospital of Jilin University in Jilin, China, the two lesions had grown in volume. Results of magnetic resonance spectroscopy and plasma 1-3-β-D-glucan activity suggested a possible fungal infection.	Cystic fluid was cultured in CHROM agar.	Itraconazole was initiated after surgery. The patient regained consciousness and regained the ability to speak.	Recovered
		*Candida* chromogenic medium and purple colony growth were present following 7 days in culture. Following 2 weeks of culture, the medium exhibited a scattered, gram-staining, oval yeast that was identified as C. glabrata.		
**Wee et al. 2021 [ 9 ].**	An elderly Singaporean Chinese female presented with a one-week history of fever.	Cultures of CSF grew heavy growth of *C. glabrata*. Antifungal susceptibilities were tested for the isolate, with the following results: minimum inhibitory concentrations for amphotericin B=0.5 mg/L; 5-fluorocytosine ≤0.06; fluconazole=2 mg/L.	The patient was started on intravenous liposomal amphotericin B at a dose of 5 mg/kg, as well as oral flucytosine at a dose of 25 mg/kg 4 times daily, for 2 weeks.	Recovered
	A CSF β-d-glucan supported the decision of treatment cessation despite incomplete resolution of CSF biochemical parameters.			

CSF: cerebrospinal fluid

In the epidemiology of *C. glabrata* infections, the primary risk factors are nosocomial settings and immunocompromised individuals. Additional specific risk factors include prolonged hospitalization, prior use of antibiotics, fluconazole treatment, and hand carriage by hospital personnel [ 10 ]. In this case report, significant risk factors, such as immunocompromised host and prior antibiotic usage, are evident due to the six-month course of anti-tubercular treatment of the patient. However, other potential risk factors, including extended hospital stays, fluconazole administration, and transmission via hand carriage by hospital staff remain uncertain.

### Therapeutic challenge

No single trial has demonstrated the clear superiority of one therapeutic agent over another. Careful analysis of the clinical data sometimes leads to conflicting conclusions. For instance, treatment of patients with candidemia via a combination of amphotericin B and fluconazole is at least as effective as using a higher dose of fluconazole alone (800 mg daily). However, this combination is seldom used in current practice since echinocandins are a safer and more effective alternative [ 11 , 12 ].

In the present report, the patient was initially administered fluconazole empirically (low dose), although later, the dose was raised. One of the known virulence factors of the *C. glabrata* complex is its intrinsic low susceptibility to azoles, especially fluconazole [ 14 ]. Generally, azoles are preferred as the initial prophylactic treatment for fungal infections due to their affordability. Despite the risk of development of cross-resistance to other azoles, they are also considered a secondary option for the treatment of invasive infections caused by various *Candida* species [ 15 ]. Moreover, the higher recommended daily dose of fluconazole is not empirically administered as a primary antifungal agent before the identification of *C. glabrata* or any other fungal pathogen [ 12 ].

In this case, despite the administration of fluconazole, amphotericin B, and flucytosine, the condition of the patient deteriorated rapidly, highlighting the high mortality associated with *C. glabrata* infection. Additionally, the immune status of the patient may have further exacerbated her mortality risk. In this case, on day 8, the patient developed complications, including mild kidney function abnormalities and hypokalemia. The observed renal abnormalities suggested the possibility of acute kidney injury, likely aggravated by amphotericin B toxicity. 

Empirical therapy with amphotericin B is especially indicated in the granulocytopenic patient with persistent fever after 3-7 days of antibiotic therapy, even in the absence of microbiological confirmation [ 5 ]. As a resource-constrained institution, the financial strain on patients was a major concern when initiating antifungals empirically. 

### Antifungal susceptibility

The AFST of the organism by disk diffusion method showed that it was only resistant to itraconazole. The Vitek-2 reports later revealed that the organism had a MIC of 2 µg/mL for both amphotericin B and flucytosine. According to Clinical and Laboratory Standards Institute breakpoints, this MIC value indicated intermediate resistance to amphotericin B and sensitivity to flucytosine for *C. glabrata*. [ 13 ]. Hence, the possibility of drug-resistant *C. glabrata* cannot be ignored.

In 2012, the initial case of fungal meningitis documented in this region involved the isolation of *Cryptococcus gatti* from a patient with AIDS [ 16 ]. Considering the small size of the region, there is a need to keep a close eye on invasive fungal infection cases in the future. 

## Conclusion

This case emphasized the challenges in the diagnosis and treatment of invasive fungal infections, particularly those caused by *C. glabrata* in immunocompromised patients. This case also highlighted the high mortality of *C. glabrata* infections, especially in patients with compromised immune systems. Possibility of nephrotoxicity contributing to the deterioration and eventual death of the patient underscored the critical need for careful monitoring of amphotericin B therapy, particularly in patients with compromised immune systems. This report emphasized the necessity for improved fungal diagnostics and the availability of a broader range of antifungal agents, including echinocandins, in resource-limited settings.
